# Thyroxin Protects White Matter from Hypoxic-Ischemic Insult in the Immature Sprague–Dawley Rat Brain by Regulating Periventricular White Matter and Cortex BDNF and CREB Pathways

**DOI:** 10.3390/ijms19092573

**Published:** 2018-08-29

**Authors:** Pi-Lien Hung, Mei-Hsin Hsu, Hong-Ren Yu, Kay L. H. Wu, Feng-Sheng Wang

**Affiliations:** 1 Department of Pediatrics, Kaohsiung Chang Gung Memorial Hospital and Chang Gung University College of Medicine, Kaohsiung 33303, Taiwan; flora1402@cgmh.org.tw (P.-L.H.); a03peggy@cgmh.org.tw (M.-H.H.); yuu2002@cgmh.org.tw (H.-R.Y.); 2 Center for Translational Research in Biomedical Sciences, College of Medicine, Kaohsiung Chang Gung Memorial Hospital and Chang Gung University, Kaohsiung 33303, Taiwan; klhwu@cgmh.org.tw; 3 Core Facility for Phenomics & Diagnostics, Department of Medical Research, Kaohsiung Chang Gung Memorial Hospital and Chang Gung University College of Medicine, No123, Rd Ta-Pei, Niao-Song District, Kaohsiung 33303, Taiwan

**Keywords:** thyroxin, brain-derived neurotrophic factor (BDNF)-TrkB signaling pathway, cAMP response element-binding protein (CREB), ischemia, immature brain injury

## Abstract

Background: Periventricular white-matter (WM) injury is a prominent feature of brain injury in preterm infants. Thyroxin (T4) treatment reduces the severity of hypoxic-ischemic (HI)-mediated WM injury in the immature brain. This study aimed to delineate molecular events underlying T4 protection following periventricular WM injury in HI rats. Methods: Right common-carotid-artery ligation, followed by hypoxia, was performed on seven-day-old rat pups. The HI pups were injected with saline, or 0.2 or 1 mg/kg of T4 at 48–96 h postoperatively. Cortex and periventricular WM were dissected for real-time (RT)-quantitative polymerase chain reactions (PCRs), immunoblotting, and for immunofluorescence analysis of neurotrophins, myelin, oligodendrocyte precursors, and neointimal. Results: T4 significantly mitigated hypomyelination and oligodendrocyte death in HI pups, whereas angiogenesis of periventricular WM, observed using antiendothelium cell antibody (RECA-1) immunofluorescence and vascular endothelium growth factor (VEGF) immunoblotting, was not affected. T4 also increased the brain-derived neurotrophic factors (BDNFs), but not the nerve growth factor (NGF) expression of injured periventricular WM. However, phosphorylated extracellular signal regulated kinase (p-ERK) and phosphorylated cyclic adenosine monophosphate response element-binding protein (p-CREB) concentrations, but not the BDNF downstream pathway kinases, p38, c-Jun amino-terminal kinase (c-JNK), or Akt, were reduced in periventricular WM with T4 treatment. Notably, T4 administration significantly increased BDNF and phosphorylated CREB in the overlying cortex of the HI-induced injured cortex. Conclusion: Our findings reveal that T4 reversed BNDF signaling to attenuate HI-induced WM injury by activating ERK and CREB pathways in the cortex, but not directly in periventricular WM. This study offers molecular insight into the neuroprotective actions of T4 in HI-mediated WM injury in the immature brain.

## 1. Introduction

Periventricular white-matter (WM) injury is a major cause of brain injury, and underlies neuromotor abnormalities in preterm infants [1]. While the etiology of this neural disorder remains inconclusive, hypoxic ischemia (HI) is a major risk factor. While term newborns with HI injury show cerebral neocortex and deep gray-matter lesions [2], cerebral WM is chiefly involved in preterm newborns with HI encephalopathy (HIE) [3]. There is increasing clinical evidence and laboratory animal studies that reveal that oligodendrocyte lineage is most vulnerable to HI injury in preterm infants [1,4]. In addition, preoligodendrocytes (pre-OLs) in the immature brain appear along with periventricular WM during a critical time period of WM injury [4]. Pre-OLs are more likely in HI-mediated dysfunction than mature oligodendrocytes, indicating that early intervention may protect pre-OL damage during HI, thus reducing the severity of WM injury in the immature brain.

The thyroid hormone (TH) triggers multiple biological activities that are essential for oligodendrocyte maturation and myelination [5]. It enhances the proliferation of committed oligodendrocyte precursors in the early development of brain tissue, and increases the morphology and function of postmitotic oligodendrocytes [6]. Transient hypothyroxinemia correlates with WM injury, cerebral palsy, and poor cognitive performance in preterm infants [7,8]. We previously demonstrated that TH rescued HI-induced WM injury in the immature brain via upregulating brain-derived neurotrophic factors (BDNFs) in periventricular WM [9]. The molecular events underlying the remedial effects of T4 on WM damage warrant investigation.

BDNF binds to the TrkB receptor, activating several intracellular signaling pathways, including phosphatidylinositol 3-kinase (PI3K) and mitogen-activated protein kinases (MAPKs), to trigger neuroprotective actions [10,11,12]. Of the MAPK members, ERK contributes to growth factor-mediated cell growth and differentiation, while JNK and p38 are involved in inflammatory cytokine-induced cell death [13,14,15]. The PI3K pathway activates Akt, which also regulates cell survival and function [16]. Accumulating evidence reveals that the PI3K pathway [17] and ERK signaling [18,19] participate in BDNF protection against HI-mediated cortical neural damage during brain development. BDNF activates PI3K signaling and promotes neuronal survival [20,21]; however, the effect of BDNF in glial cells remains elusive. We previously showed that T4 improved HI-induced pre-OLs apoptosis in the immature brain [9]. We hypothesize that BNDF, PI3K, or MAPK pathways in oligodendrocytes may contribute to the T4 attenuation of HI-induced brain injury.

This study examined whether exogenous T4 treatment can alter oligodendrocyte survival in the developing brain after HI insult, and aimed to verify the molecular events mediating T4-induced reversal of oligodendrocyte death.

## 2. Results

### 2.1. T4 Rescues HI-Mediated Hypomyelination in Injured WM

**Figure 1:**
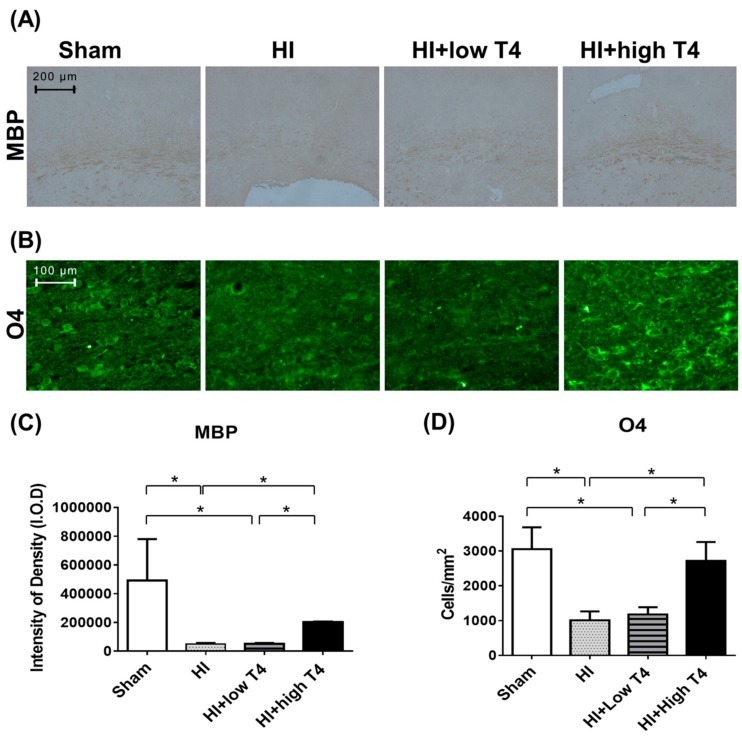
Thyroxin treatment reversed hypoxic-ischemic (HI)-induced white-matter (WM) injury. (**A**) The (HI) and HI+low thyroxin (T4) groups showed weak myelin basic-protein (MBP) immunostaining compared to the sham group, whereas the HI+high T4 group displayed strong MBP immunoreactivity. Scale bar, 200 µm. (**B**) Few positive cells for O4 immunofluorescence were observed in the HI and HI+low T4 groups. A great number of O4-immunostained cells remained in the HI+high T4 groups. Scale bar, 100 µm. (**C**) Intensity of density (I.O.D) of MBP staining in the WM. The I.O.D of MBP staining in the HI and HI+low T4 groups were significantly lower compared to the sham group, while HI+high T4 treatment attenuated the HI-induced loss. (**D**) HI reduced the number of O4-positive cells, whereas HI+high T4 treatment significantly increased the number of O4-immunostained cells compared to both HI and HI+ low T4 groups. * *p* < 0.05.

This experiment tested whether T4 treatment can restore HI-induced WM damage. WM displayed weak myelin basic-protein (MBP) immunostaining in the HI compared with the sham group (Figure 1A), with HI significantly reducing MBP-immunostaining intensity (*p* < 0.01, Figure 1C). These data suggest that HI causes hypomyelination in injured WM. It is worth noting that reduced MBP immunoreactivity was significantly reversed in HI-injured tissue treated with 1 mg/kg T4 (*p* < 0.05, Figure 1A,C). With respect to the priming of oligodendrocyte precursors, WM tissue displayed weak O4 immunofluorescence, along with a significant reduction in the number of O4-immunostained cells in the HI group compared to the sham group (*p* < 0.05, Figure 1B,D). Administration of 1 mg/kg T4 improved O4 immunofluorescence and significantly increased the number of O4-immunostained cells in injured WM compared to the HI and sham groups (*p* < 0.05, Figure 1B,D). Treatment with 0.2 mg/kg T4 had no effect on the number of MBP- or O4-immunostained cells following HI (Figure 1C,D).

### 2.2. T4 Promoted MBP 23KDa Isoform Expression in Injured WM

**Figure 2:**
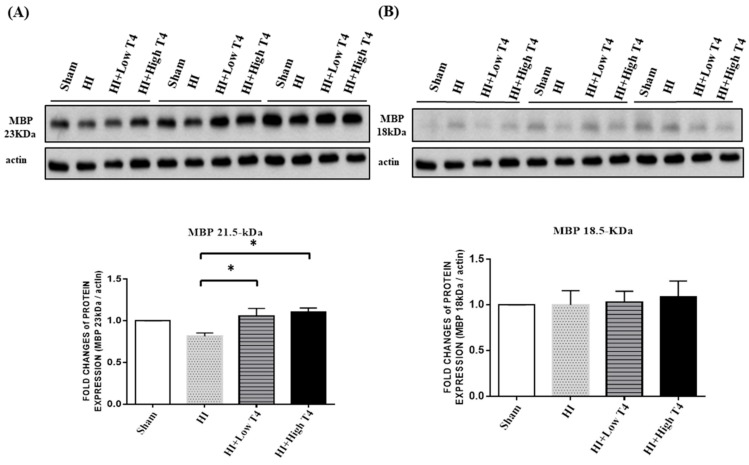
Immunoblots of MBP isoforms in injured WM. (**A**) Both low and high doses of T4 increased the levels of the 21.5 kDa isoform. (**B**) Neither dose of T4 had an effect on the levels of the 18.5 kDa isoform. * *p* < 0.05.

MBP contains isoforms ranging from 14–21.5 kDa through transcriptional splicing reactions. Each plays a distinct role in oligodendrocyte development. Of the isoforms, the 18.5 kDa MBP in the cytoplasmic compartment is the most abundant in mature central nervous system myelin [22]. The 21.5 kDa MBP exists in the nuclei and cytoplasm of oligodendrocytes during active myelination [23]. Experiments were performed to investigate whether either isoform plays a role in injured WM upon treatment with T4. Of interest, two MBP bands corresponding to 21.5 kDa and 18.5 kDa existed in the WM specimens. T4 treatment at both doses significantly increased the 21.5 kDa MBP in injured WM (Figure 2A), whereas the levels of the 18.5 kDa MBP were unchanged (Figure 2B). These results suggest that T4 increases myelination in injured WM. * *p* < 0.05.

### 2.3. T4 Restored BDNF But Not NGF Expression of Injured WM Tissues

**Figure 3:**
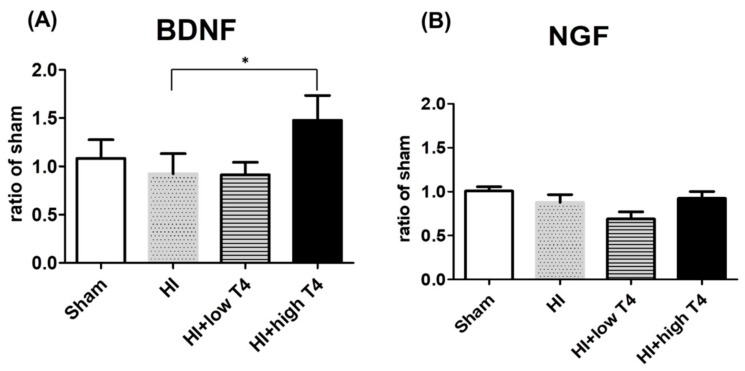
Thyroxin treatment upregulated brain-derived neurotrophic-factor (BDNF) expression in injured WM. (**A**) BDNF mRNA expression in WM was significantly upregulated in the HI+high T4 group compared to the HI group; (**B**) nerve growth factor (NGF) mRNA expression in WM was not significantly different between the HI and HI+T4 groups. * *p* < 0.05.

Neurotrophic factors are important to oligodendrocyte survival [24], and BDNF has been found to promote myelin basic-protein accumulation [25]. We therefore examined whether T4 treatment could alter BDNF or NGF expression in injured WM. RT-PCR analyses showed that HI had no effect on BDNF or NGF expression in WM. Of interest, BDNF, but not NGF expression, was significantly increased with 1 mg/kg T4 treatment compared with the HI group (*p* < 0.05, Figure 3A,B).

### 2.4. TrkB, Atk, p38, or JNK Did Not Actively Respond to T4 Treatment

**Figure 4:**
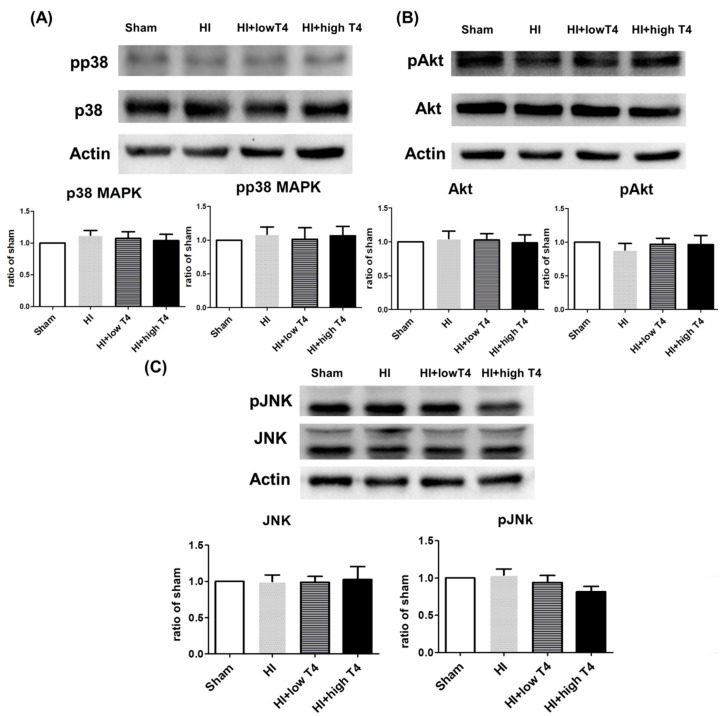
Effects of HI insult and T4 treatment on p38 mitogen-activated protein kinases (MAPK), AKT, and JNK signaling pathways. HI and HI+T4 had no effect on the concentrations of (**A**) total p38 and phosphorylated p38 MAPK, (**B**) total Akt and phosphorylated Akt, or (**C**) total JNK and phosphorylated JNK.

BDNF binds to the TrKB receptor [26], which triggers downstream signaling pathways, including MAPK, PI3K, and PLC [27,28,29,30], to regulate oligodendrocyte function. We therefore investigated whether MAPK or Akt signaling affects periventricular oligodendrocytes with HI and HI+T4 treatment. The MAPK pathways include the extracellular signal-regulated kinase (ERK), c-Jun amino-terminal kinase (JNK), and p38 signaling cascades. These pathways are involved in various cellular responses, including growth, proliferation, and survival [31,32]. We therefore examined JNK, p38, ERK, and Akt signaling in the following experiments. Unexpectedly, neither total nor phosphorylated p38, JNK, or Akt concentrations were significantly affected following HI compared to the sham group. T4 treatment also had no effect on the total or phosphorylated forms of these molecules (Figure 4A–C).

### 2.5. T4 Reduced HI-Induced ERK Activation and CREB Phosphorylation

**Figure 5:**
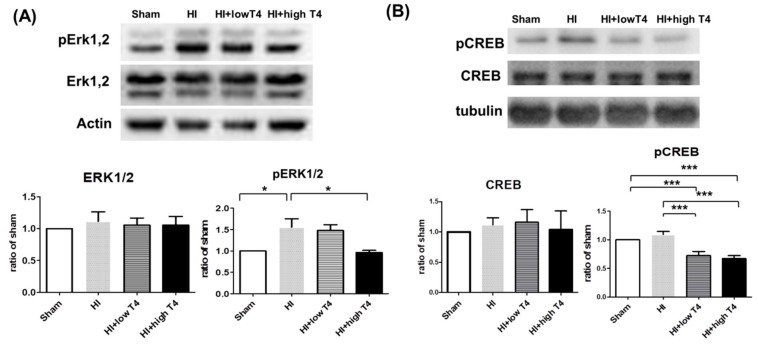
Effects of HI insult and T4 treatment on cyclic adenosine monophosphate response element-binding protein (CREB) and extracellular signal regulated kinase (ERK) signaling in WM. (**A**) HI insult increased the levels of phosphorylated ERK1/2, whereas the high dose of T4 significantly attenuated the HI enhancement of phosphorylated ERK1/2 levels in WM. (**B**) HI had no effect on CREB levels, while T4 treatment significantly decreased the levels of phosphorylated CREB (* *p* < 0.05; *** *p* < 0.005).

ERK participates in BDNF-mediated neuroprotection during HI-induced injury in the developing brain [18]. It also mediates BDNF-regulation of CREB phosphorylation [17]. Thus, we investigated the effect of T4 on ERK and CREB activation in HI-induced injured tissue. HI significantly increased phosphorylated ERK2 but not phosphorylated ERK1 levels or total ERK concentrations, and significant increased phosphorylated CREB levels. T4 treatment significantly attenuated the HI-induced elevations of phosphorylated ERK1 and phosphorylated CREB (Figure 5A,B).

### 2.6. T4 Did Not Change Neointimal Formation in HI-Injured WM

**Figure 6:**
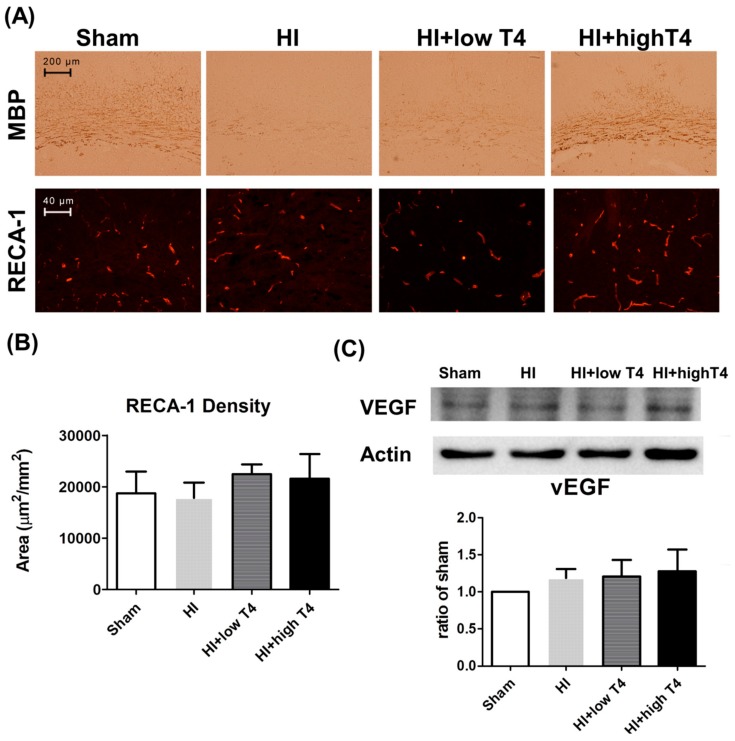
T4 treatment restored myelination but did not alter neovascular remodeling following WM injury. (**A**) A high dose of T4 treatment enhanced myelination, as evident from MBP staining, whereas antiendothelium cell antibody (RECA-1) staining, a marker of neovascular angiogenesis, was similar across groups. Scale bar, 200 µm. (**B**) HI or HI+T4 treatment had no effect on RECA-1 levels. Scale bar, 40 µm. (**C**) HI or HI+T4 treatment had no effect on vascular endothelium growth factor (VEGF) levels.

Cortical gray matter also participates in neurovascular matrix remodeling upon brain injury [33,34,35]. Using immunofluorescence analysis of the blood vessel-lining marker RECA-1, we further tested whether HI or T4 treatment alters neovascular formation in injured WM. Although injured WM displayed weak MBP immunostaining, a strong RECA-1 immunofluorescence reaction was observed in all groups (Figure 6A). Neither HI nor HI+T4 treatment affected RECA-1 immunostaining density or angiogenic growth-factor VEGF concentrations (Figure 6B,C).

### 2.7. T4 Increased BDNF Expression in Cortical Neurons

**Figure 7:**
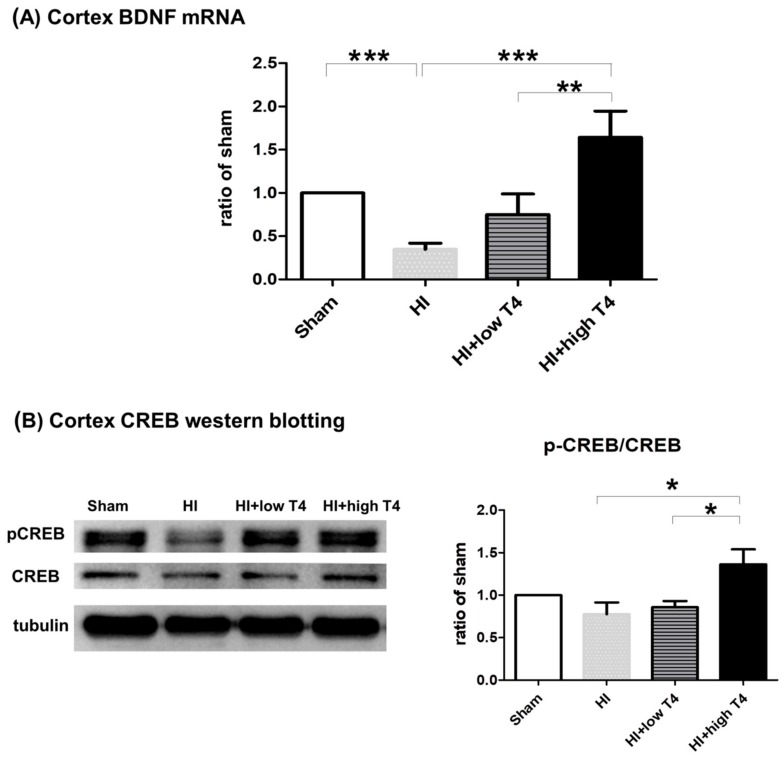
T4 treatment increased BDNF expression in cortical neurons. (**A**) HI significantly decreased BDNF mRNA expression in the cortex, whereas a high dose of T4 attenuated HI-induced loss of BDNF expression. (**B**) Concentrations of phosphorylated CREB were significantly increased in the cortex with a high dose of T4 treatment. * *p* < 0.05; ** *p* < 0.01; *** *p* < 0.005.

Since BDNF also acts as a protective factor against hypoxic damage in neurons [36], we examined the effect of T4 on BDNF levels in the cortex following WM injury. HI significantly decreased BDNF mRNA expression in the cortex (Figure 7A). Cortical BDNF mRNA expression (Figure 7A) and phosphorylated CREB concentrations (Figure 7B) were significantly elevated with high-dose T4 treatment compared with the HI group (Figure 7A). Our data suggest that cortical BDNF is involved in the T4-mediated attenuation of HI-induced WM injury.

## 3. Discussion

The present study revealed that HI causes periventricular WM injury and induces the death of pre-OLs. T4 treatment reversed the WM injury and death of pre-OLs by upregulating BDNF in both the cortex and WM compartments without changing the neurovascular unit. While downstream effects of BDNF on the CREB pathway warrant further characterization, the current results allude to the importance of an intact neurovascular unit to bridge BDNF signaling between the cortex and white matter.

It is well known that hypothyroxinemia contributes to cognitive underperformance in preterm infants. Periventricular WM injury correlates with aberrant neuromotor function. While T4 administration promotes neural-cell survival, its remedial actions in HI-induced WM injury remain elusive. The current results show that a 1 mg dose of T4 ameliorates the HI-induced loss of oligodendrocytes. Increased BDNF and CREB phosphorylation contribute to the T4 protection against myelination loss in HI-injured WM. T4 also upregulates BDNF and CREB signaling in cortical neurons. This study is the first to explore thyroid-hormone function in developing WM upon HI injury and the molecular events by which T4 attenuates HI-induced brain injury, underpinning possible remedial actions of T4 in neural dysfunction of the developing brain.

Previous studies have demonstrated that BDNF activates various signaling pathways; its actions depend on cell type, growth conditions, and deleterious stresses [17]. In this study, the downstream effects of BDNF on CREB were activated in HI-injured WM. Our results are in agreement with previous studies showing that CREB phosphorylation in oligodendroglia can be upregulated throughout the development period, whereas MAPK-dependent phosphorylation appears to be downregulated in mature OLGs [37,38]. Importantly, T4 reduced the HI-induced ERK and CREB phosphorylation of injured WM. ERK signaling actively responded to HI and T4 modulation of myelination in WM. ERK mediated the BDNF modulation of CREB phosphorylation of cultured cortical neurons, rather than developmental oligodendrocytes. T3 inhibits CREB phosphorylation at Ser133 and decreases CRE-promoter activity and transcription in pituitary cells [39]. Thyroid-hormone-receptor signaling is found to reduce the transcription of CREs genes [40]. We speculate that different brain-injury types may confer various intracellular signaling transductions. In addition, the cerebral endothelium is abundant in factors that exert endogenous neuroprotection [41,42]. Loss of vascular neuroprotection is linked to the conditions of stroke, brain trauma, and neurodegeneration [43,44,45].

VEGF-A signaling triggers CREB phosphorylation, protecting neurons and cerebral vascular endothelial cells [46]. The thyroid hormone regulates cerebral vascular complexity, density, and microvessel diameter during CNS development in vitro and in vivo. In this study, HI or T4 treatment did not significantly affect neurovascular activity, as evident from strong RECA-1 and VEGF signaling in injured WM. Together, our collective results and those of others show that multiple pathways actively respond to T4 treatment and sustain various biological reactions, including myelination and neurovascularization, to attenuate HI-mediated WM injury. The substantial neurovascular effects in injured WM also suggest that intact neurovascular networks may bridge the BDNF signaling paths between periventricular WM and the cortex upon T4 injection [47].

We acknowledge the limitations of our studies. We do not exclude the possibility that T4 may change cortical function to directly or indirectly protect WM. While the neurovascular unit is an important target of brain injury, the interplay between the central nervous and vascular systems is required to maintain blood–brain-barrier (BBB) integrity and promote neural function and regeneration [48]. In this study, intact neurovascular tissue presumably affected BDNF signaling between WM and the cortex. The molecular events underlying the crosstalk between the cortex, white matter, and BBB integrity in T4-mediated protection against HI-induced brain injury warrants further elucidation.

## 4. Materials and Methods

### 4.1. Ischemia and Hypoxia-Induced in Premature Brains in Rat Pups

All procedures were approved by and in compliance with the guidelines of the Institutional Animal Care and Use Committee. Litters of Sprague–Dawley rat pups were raised with dams in an air-conditioned environment with a 12 h light/dark cycle. The male pups (seven days old) were anesthetized with 2.5% halothane balanced with cabinet air, and the right common carotid artery was permanently ligated. One hour after resuscitation, the pups were placed in a thermostatic (37 °C) and airtight chamber (500 mL) circulated with humidified oxygen (3 L/min, 6.5% O_2_) for one hour.

### 4.2. T4 Administration

Animals were randomly divided into 4 groups by a research assistant blind to the experiments. To determine the sample size, we set a power value of around 90%, and we also determined the direction of the effect as two tailed with α level = 0.05, δ mean = 1.2, σ2 = 0.64, and attrition around 1%. We then calculated the sample size using the following formula: N = 2σ2(Zα + Z1 − β)2/(μ1 − μ2)2 = 9.33.

Note: σ2 = 0.64; (μ1 − μ2)2 = δ mean = 1.2; Zα = 1.96; Z1 − β = 1.28, which were determined by a standard normal distribution table.

The corrected sample size = sample size/(1% attribution of animal) = 9/0.9 = 10. However, statistical significance was observed with 9 pups, so we used only 9 rat pups for each group in this experiment. Eight rats were treated with 0.2 mg/kg L-thyroxin (T4; Sigma, Kawasaki, Japan) (HI+low T4 group), 9 rats were administered 1 mg/kg T4 (HI+high T4 group), and 9 animals were injected with normal saline (NS). T4 or NS was injected intraperitoneally 48 to 96 h after hypoxia. Eight pups received a sham operation (sham controls). While T4 treatment reduced body weight, there was no significant difference between groups on postnatal day (P) 7, P9, and P11.

### 4.3. Immunohistochemistry

Sections of brain tissue were deparaffinized in graded alcohol solutions and xylene. The sections were then blocked with 3% H_2_O_2_ (37 °C, 30 min) and goat serum (37 °C, 60 min), followed by the addition of the rabbit antirat monoclonal anti-MBP (1:500, Chemicon, Nippon Chemi-Con, Taiwan). Sections were incubated overnight at 4 °C, followed by incubation with a biotin-labeled goat antirabbit IgG secondary antibody at 37 °C for 60 min. The negative control sections were incubated with phosphate-buffered saline (PBS) instead of with primary antibodies. All sections were then incubated with an avidin–biotin complex (37 °C, 60 min, Vector Laboratories, Burlingame, CA, USA), stained with 3′-diaminobenzidine (DAB), and dehydrated, vitrified, and mounted. Coronal sections in the region of the mid-dorsal hippocampus were examined. Stereotaxic coordinates were 2.8–3.1 mm from bregma and 2.6–3.0 mm lateral to midline [49]. Sections were immunostained for MBP to evaluate WM loss.

### 4.4. Assessment of Immunohistochemical Staining

As described previously [50], the number of O4-positive cells and the integrated optical density (IOD) of MBP signals were analyzed with imaging software (ImagePro Plus 6.0, Media Cybernetics, Silver Spring, MD, USA) at 400× magnification for O4 and 200× magnification for MBP. Three fields in the medial, middle, and lateral areas of WM in each hemisphere of each section, and four sections of each animal, were randomly selected for analysis. Four sections per brain, two at the level of the striatum (0.26 mm and 0.92 mm posterior to bregma) and two at the dorsal hippocampus (3.14 mm and 4.16 mm bregma), were chosen for this experiment based on a previous study [51]. The mean IOD values in the ipsilateral WM of each experimental group were compared with those of the control group to obtain the relative IOD ratios.

### 4.5. Quantitative Real-Time Polymerase Chain Reaction (qRT-PCR)

BDNF mRNA expression was quantified using qRT-PCR protocols, as described previously [52]. Briefly, total RNA was isolated using a RNase Mini Kit (catalog #74104, Qiagen, Dusseldorf, Germany) from thin coronal brain sections near the midseptal nucleus. cDNA was prepared using Superscript II RT (catalog #05081955001, Roche, Basel, Switzerland) and SYBR green (catalog #04913850001, Roche), and amplified with an ABI Prism 7900HT Sequence Detection System (Applied Biosystems, Foster City, CA, USA). Primers for BDNF (sense 5′-AGCTTCATTCTGAGAGACG-3′; antisense 5′-GTCAACATAAACCACCGACA-3′) and the housekeeping gene GAPDH (accession number: NM_001082253.1) (sense: 5′-GCGTGAACCACGAGAAGTAT-3′; antisense: 5′-CCTCCACAATGCCGAAGT-3′) were used. Primer specificity was validated by melt-curve analysis. The Ct value of fluorescence units was computed automatically. Relative BDNF mRNA expression was calculated as normalized GADPH expression.

### 4.6. Immunofluorescence Staining

To assess the distribution and morphology of vascular tissue in periventricular WM, one series of cryostat brain sections (1 in 3, 60 µm apart) was stained with an antibody raised against rat endothelium. Sections of brain tissue were infiltrated with 4% paraformaldehyde, soaked in 3% paraformaldehyde for 3 h at 4 °C, cryoprotected in 30% sucrose for 12 h at 4 °C, and frozen at −80 °C. The frozen sections (20 μm thick) were air-dried and washed 3 times with PBS, incubated with 0.5% Triton X-100 for 5 min at room temperature, and again washed 3 times with PBS. The sections were then blocked with 10% goat serum for 30 min at 37 °C, followed by incubation with the mouse monoclonal primary antibody anti-RECA-1 (1:100, Abcam, San Francisco, CA, USA) overnight at 4 °C. Sections incubated in the absence of primary antibodies served as negative controls. Tissue sections were washed 4 times with PBS-Triton X-100 incubated for 60 min at 37 °C with the secondary antibody conjugated with Alexa Fluor 488. The tissue sections were washed 3 times with PBS and the nuclei were stained for 2 min with 4′,6-diamidino-2-phenylindole (DAPI, 1:1000 dilution (Sigma-Aldrich, Santa Clara, CA, USA). Following additional washes, images of the tissues were captured by a fluorescent microscope.

### 4.7. Morphometric Analysis

For each animal, sections of ipsilateral WM were selected and digitized with a digital camera connected to a Nikon fluorescent microscope (Nikon Eclipse E400 Epi-Fluorescence Microscope, Tokyo, Japan) with a 10 objective. Four sections per brain, 2 at the level of the striatum (0.26 mm and 0.92 mm posterior to the bregma) and 2 at the dorsal hippocampus levels (3.14 mm and 4.16 mm posterior to the bregma) [53] were selected for RECA-1 densinometry and vascular-length measurements. The area of blood vessels stained with RECA-1 within these selected regions was measured and analyzed using Image Pro Plus 6.0. The values are expressed as the total area of blood vessels per mm^2^ (µm^2^/mm^2^). The blood vessels assessed included arteries, arterioles, capillaries, and venules. Data are presented as mean ± standard error of mean (SEM). The quantitative data were analyzed by one-way analysis of variance (ANOVA) followed by a Bonferroni test. A probability level of <0.05 was considered statistically significant.

### 4.8. Western Blotting

P7 rats were subjected to unilateral common-carotid-artery ligation (UCL)-hypoxia (6.5% O2), followed by IP administration of normal saline or 1mg/kg T4 on P7, P9, and P11. The frozen periventricular WM was dissected at P11 and homogenized in a sample buffer (3% SDS, 10% glycerol, and 62.5 mM Tris-HCl) using a mechanical homogenizer, followed by sonication and centrifugation. Protein concentrations of supernatant were determined using a BCA protein-assay kit (Pierce Kit #23227, Thermo Scientific, Waltham, MA, USA) with bovine-serum albumin to plot a standard curve. After denaturing in Laemmli buffer (catalog #161-0737, Bio-Rad, Hercules, CA, USA), equal amounts of protein (10–20 μg) were loaded onto 4–15% or 4–20% gradient precast gels (Bio-Rad), depending on the molecular weight of the target protein. Separated proteins were transferred onto poly-vinylidene difluoride membranes. Membranes were incubated overnight with VEGF (1:1000, Santa Cruz Biotechnology) and MBP primary antibodies (1:5000, Abcam, Cambridge, UK, catalog #ab40390). We detected proteins of interest with a chemiluminescence ECL system (GE Healthcare, Chicago, IL, USA) using secondary antibodies conjugated with horseradish peroxidase (Jackson ImmunoResearch, West Grove, PA, USA). The blots were stripped with buffer (2.5% SDS, 0.7% 2-mercaptoethanol, 62.5 mM Tris-HCl, pH 6.8) and incubated with the β-actin antibody (catalog #A5316, Sigma), followed by a secondary antibody and visualized with the chemiluminescence ECL system. The blots from each experiment were densitometrically analyzed using Image J. OD values, which were normalized to β-actin, and graphs are presented as “adjusted OD”. The adjusted OD measurements were normalized such that mean values of normal periventricular white-matter samples were equal to one, and graphs are presented as “relative OD”.

### 4.9. Statistical Analysis

Statistical analysis was performed using SPSS software version 18.0 (SPSS, Inc., Chicago, IL, USA). Continuous data are presented as mean ± SEM. Statistical significance (*p* < 0.05) was verified using one-way ANOVA along with the Tukey method for post hoc comparisons.

## 5. Conclusions

The current findings demonstrate that T4 treatment attenuates HI-induced white-matter injury and regulates BDNF and CREB signaling within periventricular WM and the cortex in the presence of an intact neurovascular network. This study also highlights the neuroprotective effects of T4 therapy during WM injury in the premature brain.
